# SARS-CoV-2 infection and disease outcomes in non-human primate models: advances and implications

**DOI:** 10.1080/22221751.2021.1976598

**Published:** 2021-09-17

**Authors:** Lunzhi Yuan, Qiyi Tang, Huachen Zhu, Yi Guan, Tong Cheng, Ningshao Xia

**Affiliations:** a State Key Laboratory of Molecular Vaccinology and Molecular Diagnostics, National Institute of Diagnostics and Vaccine Development in Infectious Diseases, School of Life Sciences, School of Public Health, Xiamen University, Xiamen, People’s Republic of China; b Department of Microbiology, Howard University College of Medicine, Washington, DC, USA; c State Key Laboratory of Emerging Infectious Diseases, The University of Hong Kong, Hong Kong, People’s Republic of China; d Joint Institute of Virology (Shantou University and The University of Hong Kong), Guangdong-Hongkong Joint Laboratory of Emerging Infectious Diseases, Shantou University, Shantou, People’s Republic of China; e Research Unit of Frontier Technology of Structural Vaccinology, Chinese Academy of Medical Sciences, Xiamen, People’s Republic of China

**Keywords:** SARS-CoV-2, non-human primates, severe acute respiratory syndrome, immunopathogenesis, vaccine and drug discovery

## Abstract

SARS-CoV-2 has been the causative pathogen of the pandemic of COVID-19, resulting in catastrophic health issues globally. It is important to develop human-like animal models for investigating the mechanisms that SARS-CoV-2 uses to infect humans and cause COVID-19. Several studies demonstrated that the non-human primate (NHP) is permissive for SARS-CoV-2 infection to cause typical clinical symptoms including fever, cough, breathing difficulty, and other diagnostic abnormalities such as immunopathogenesis and hyperplastic lesions in the lung. These NHP models have been used for investigating the potential infection route and host immune response to SARS-CoV-2, as well as testing vaccines and drugs. This review aims to summarize the benefits and caveats of NHP models currently available for SARS-CoV-2, and to discuss key topics including model optimization, extended application, and clinical translation.

## Introduction

Following the past two pandemics of beta-coronavirus infection, severe acute respiratory syndrome coronavirus (SARS-CoV) in 2002 and middle east respiratory syndrome coronavirus (MERS-CoV) in 2012, a third pandemic caused by SARS-CoV-2 has been affecting more than 200 countries with more than 200 million cases and over 4 million deaths. Most SARS-CoV-2-infected individuals exhibit mild to moderate symptoms, but approximately 20% of cases progress to severe pneumonia with respiratory distress, septic shock and/or multiple organ failures [1,2]. Currently approved clinical treatments cannot fully suppress viral replication and inflammation to rescue organ failure [3]. Despite having high genomic homology with SARS-CoV, SARS-CoV-2 is much more contagious with an *R*_0_ of 5.1∼5.7 [4] than SARS-CoV with an *R*_0_ of 3.1 [5]. Therefore, it is imminent to obtain drugs against SARS-CoV-2 infection. However, many fundamental questions of SARS-CoV-2 biology and pathology remain unanswered. For example, how does SARS-CoV-2 infection cause complicated immunopathogenesis and hyperplastic lesions in the respiratory system? What are the potential routes for SARS-CoV-2 infection and what does determine tissue tropism among digestive, cardiovascular, urinary, reproductive, and central nervous systems? Moreover, the imbalanced and complicated host immune responses might drive different outcomes of SARS-CoV-2 infection [6], which may impose a challenge for the development of vaccine and immunotherapy. All the above-mentioned biological aspects need an appropriate animal model to investigate.

**Figure 1.**
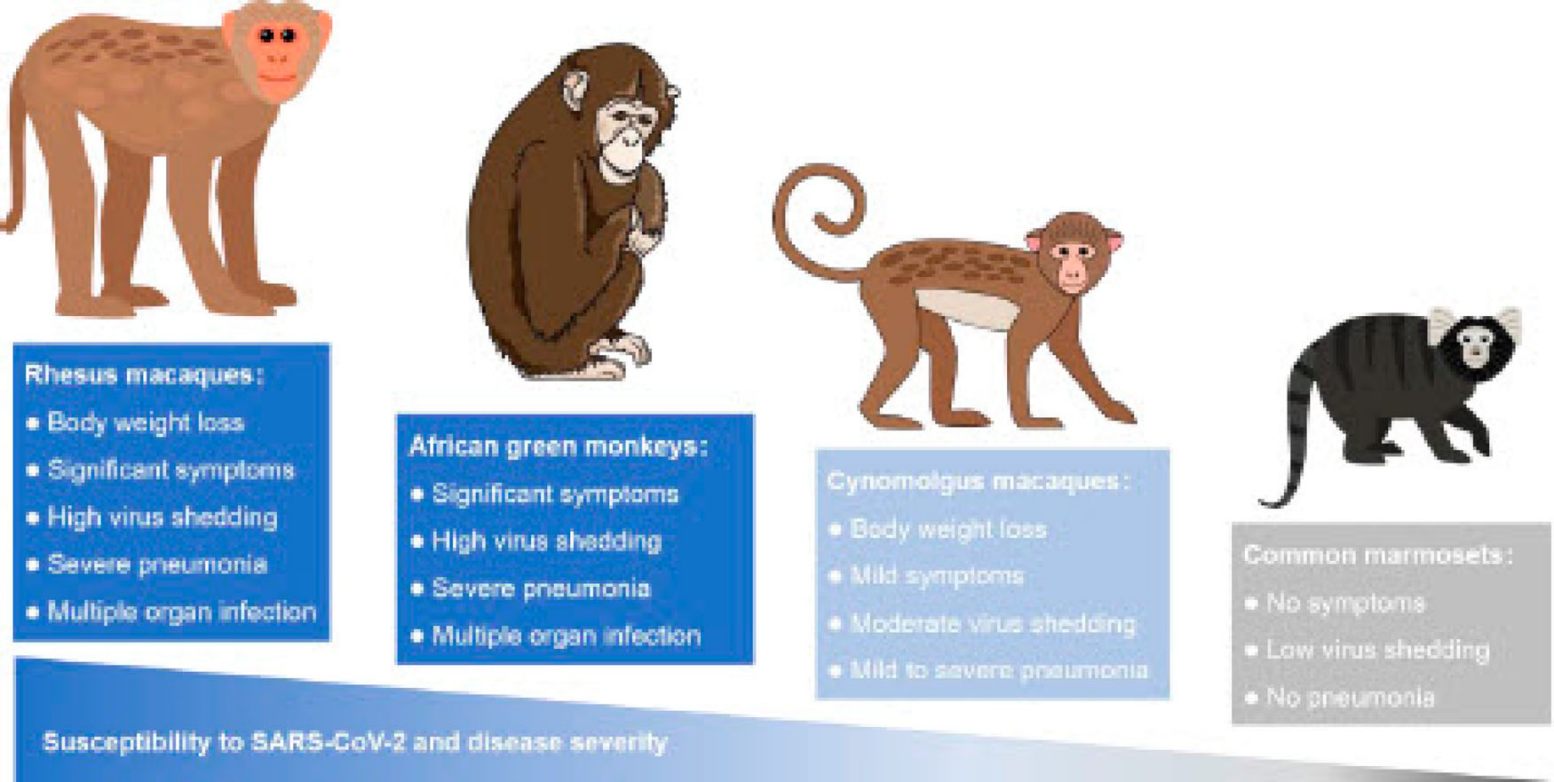
Summary of SARS-CoV-2 infection in four NHP models. SARS-CoV-2 susceptibility and disease severity of rhesus macaques (*Macaca mulatta*), cynomolgus macaques (*Macaca fascicularis*), common marmosets (*Callithrix jacchus*), and African green monkeys (*Chlorocebus sabaeus*).

An appropriate animal model is essential for pre-clinical evaluation of the safety and effect of drugs or vaccines [7]. The evolutional, anatomical, physiological, and immunological similarities of NHP to humans make the NHP an ideal model to study the pathogenesis of SARS-CoV-2 infection in humans. Recently, four old- and new-world monkeys including rhesus macaques (*Macaca mulatta*), cynomolgus macaques (*Macaca fascicularis*), common marmosets (*Callithrix jacchus*), and African green monkeys (*Chlorocebus sabaeus*) have been demonstrated permissive for infection of SARS-CoV-2 (Figure 1). Typical clinical symptoms, virus shedding, tissue lesions, and host immune responses that are similar to human patients were observed in these NHP models. Although the disease severity varies among species and individuals, the NHP models are important for both the fundamental research, vaccine, and drug discovery of SARS-CoV-2. Here, we will discuss the critical findings and implications that might guide the fundamental studies, clinical management, urgent treatment, and future public health strategy for SARS-CoV-2 infection.

## Infection route and tissue tropism of SARS-CoV-2 in NHP

The receptor-mediated entry is the first step of a viral infection in the host cell [8]. Human angiotensin-converting enzyme 2 (hACE2) has been demonstrated to be the receptor for SARS-CoV-2 [9]. It was revealed by single-cell RNA sequencing that hACE2 is highly expressed not only in respiratory tract and lung [10], but also in other tissues and organs such as testis, liver, kidney, pancreas, small intestine, and bladder [11], implying that many organs are susceptible to SARS-CoV-2 infection. In addition, cholangiocytes [12], T-lymphocytes [13], small intestine enterocytes [14], and nasal epithelial cells [15] were reported to be permissive for direct SARS-CoV-2 infection. However, many of these claims were made based on the experiments using clinical samples, or cellular and organoid models and need to be confirmed in the animal models. Because hACE2 has many important physiological functions, it is not appropriate to choose hACE2 as a candidate molecule for the development of SARS-CoV-2 vaccine or therapeutics.

SARS-CoV-2 successfully infects the NHP through the ocular conjunctival, intratracheal, intranasal, and olfactory routes [16–19]. SARS-CoV-2 infection causes relatively severe symptoms in the rhesus macaque and the African green monkey among the four NHP models. In the rhesus macaque model, combined inoculation of SARS-CoV-2 intranasally, intratracheally, and orally results in more severe diseases than those with a single inoculation route [17]. Approximately 10% body weight loss was detected from 5 to 10 days post-infection (dpi) [17,20]. Generally, the rhesus macaque and the African green monkey are more susceptible to SARS-CoV-2 infection as compared to the cynomolgus macaque and the common marmoset (Figure 1). Although varied degrees of symptoms were observed and different viral loads were detected among different ages and genders, larger group size is needed to draw a certain conclusion. In addition to the different strains of SARS-CoV-2, the infection doses or routes of viruses and ages of the NHP might also lead to the variations of viral shedding, tissue viral load, and symptoms (Table 1). Similar to those in humans, symptoms including fever, cough, irregular respiration, and abnormal chest radiography were observed in the NHPs with SARS-CoV-2 infection. Virus shedding was detectable in nose, throat, and anal swabs, bronchi-alveolar lavages [21], blood [17], as well as hand and drinking nipple swabs [22]. Viral RNA was detected in the nose, pharynx, respiratory tract, lung, gut (duodenum, jejunum, ileum, caecum, and colon), and lymphatic system [18,19]. Furthermore, viral antigens were positive in nasal turbinate, lung, stomach, gut, and mediastinal lymph node. However, no SARS-CoV-2 RNA or antigen is identified in bone marrow, the reproductive tract, and the central nervous system up to date [21]. Taken together, this information suggested a productive SARS-CoV-2 infection in NHPs. Future studies need to demonstrate whether asymptomatic infection, viral rebound, close-contact, and fecal-oral transmission of SARS-CoV-2 are possible using the NHP models.

**Table 1.** Detailed information of representative NHP models for SARS-CoV-2 infection and pathogenesis.

Research Groups and NHP species	Deng et al.	Deng et al.	Shan et al.	Munster et al.	Rockx et al.	Lu et al.			Woolsey et al.
	Rhesus macaques				Cynomolgus macaques	Rhesus macaques	Cynomolgus macaques	Common marmosets	African green monkeys
Age	3∼5 years	3∼5 years	6∼12 years	Adult	Young (4∼5); Old (15∼20)	Young; Adult; Old	Adult	Adult	Adult
Amount	3	4	6	8	4	12	6	6	6
Gender	Male	–	Male (3); Female (3)	Male (4); Female (4)	–	Male (6); Female (6)	Male (3); Female (3)	Male (3); Female (3)	Male (2); Female (4)
Viral strain	SARS-CoV-2/WH-09/human/2020/CHN (Wuhan, China)		IVCAS 6.7512 (Wuhan, China)	nCoV-WA1-2020 (USA)	Isolation from a German traveller returning from China	Local isolation (Guangdong, China)	Local isolation (Guangdong, China)	Local isolation (Guangdong, China)	Isolation from an Italy traveller returning from China
Infection dose	1 × 10E6 TCID50		7 × 10E6 TCID50	2.5 × 10E6 TCID50	–	4.75 × 10E6 PFU for adult and old; Half dosage for young	4.75 × 10E6 PFU	1 × 10E6 PFU	4.6 × 10E5 PFU
Infection route	Ocular conjunctival (2) or intratracheal (1)	Intratracheal	Intratracheal	Combination of intranasal (0.5 mL per nostril), intratracheal (4 mL), oral (1 mL) and ocular (0.25 mL per eye)	Combined intratracheal and intranasal	Combination of intranasal (0.5 mL), intratracheal (4 mL) and oral (0.25 mL) for adult and old	Combination of intranasal (0.5 mL), intratracheal (4 mL) and oral (0.25 mL)	Intranasal	Combination of intratracheal and intranasal routes
Positive swabs and biological samples	Nose, throat and anal (1∼7 dpi)	Nose, throat and anal (1∼14 dpi)	Nose, throat and anal (1∼14 dpi)	Nose, throat and anal (1∼20 dpi)	Nose, throat and anal (1∼8 dpi)	Nose, throat and anal (1∼14 dpi)	Nose, throat and anal (1∼14 dpi)	Nose, throat and anal (1∼12 dpi)	Nose (0∼12 dpi; 35∼40 dpi), oral (0∼7 dpi;), rectal (0∼15 dpi), bronchoalveolar lavage fluid (0∼7 dpi)
Peak tissue viral load	> 1 × 10E7 viral RNA copies in upper left lung at 7 dpi (intratracheal)	> 1 × 10E8 viral RNA copies in nose turbinate at 7 dpi	> 1 × 10E7 viral RNA copies in lower right lung at 3 dpi	> 1 × 10E8 viral RNA copies in right and left lung at 3 dpi	1 × 10E4 TCID50eq in lung at 4 dpi	> 1 × 10E7 viral RNA copies in rectum at 7 dpi	> 1 × 10E3 viral RNA copies in spleen at 13 dpi	Undetectable (13 dpi)	> 1 × 10E7 viral RNA copies in lung at 5 dpi
Clinical illness	Increased body temperature (1/3)	200∼400 g weight loss from 5∼15 dpi; reduced appetite, increased respiration rate, and hunched posture were transient after the initial challenge; Chest X-ray at 7 dpi showed that the upper lobe of the right lung had varying degrees of the localized infiltration and interstitial markings	7∼8% weight loss at 14 dpi in two animals; Chest X-ray at 7 dpi showed signs of interstitial infiltrates and pneumonia; A variable degree of consolidation, oedema, haemorrhage, and congestion in bright red lesions throughout the lower respiratory tract and right lung at 6 dpi	5∼10% weight loss from 5∼15 dpi in two animals; Reduced appetite, hunched posture, pale appearance, dehydration and irregular respiration patterns; Chest X-ray showed pulmonary infiltrates in one animal in 1∼12 dpi; Multifocal and random hilar consolidation and hypaeremia at 3 and 21 dpi	Two animals had foci of pulmonary consolidation in lung at 4 dpi; a serous nasal discharge in one aged animal at 14 dpi	Increased body temperature (12/12); Chest X-ray abnormality; > 10% weight loss at 10 dpi (8/12); severe pneumonia; inflammation in liver and heart	Increased body temperature (2/6); Chest X-ray abnormality; one animal showed 10% weight loss at 10 dpi; mild to severe pneumonia; inflammation in liver and heart	No symptoms	Fever, decreased appetite, and nasal exudate from 0 to 7 dpi; intermittent symptoms were observed from 8 to 57 dpi

## Immunopathology and hyperplastic lesions in the target organs of SARS-CoV-2-infected NHPs

Diverse degrees of trachea and lung lesions were observed in SARS-CoV-2-infected NHPs. The lesions were multifocal, from mild-to-moderate, interstitial pneumonia that frequently centred on terminal bronchioles [22,23]. The pneumonia was characterized by diffusive haemorrhage, inflammatory infiltration, consolidation, and hyperaemia. The normal alveolar structure was impaired with thickening of alveolar septate. The alveoli were filled with cell debris, inflammatory cells including neutrophils, erythrocytes and macrophages, oedema fluid and fibrin with formation of hyaline membranes [22]. Remarkably, epithelial cell originated syncytium was observed in the alveolar lumen [22]. Ultrastructural analysis of lungs further confirmed the abnormality of lung cells after SARS-CoV-2 infection [17,21]. In addition, macrophages, type I (flat), and type II (cuboidal) pneumocytes in the affected lung tissues were demonstrated positive for SARS-CoV-2 antigens [21,22]. Interstitial pneumonia was presented in all the four NHP models but the diffusive alveolitis and more severe lung injury were seen only in the rhesus macaque and the African green monkey. Meanwhile, SARS-CoV-2 antigen expression, inflammatory infiltration, tissue impair, and hyperplasia were also detectable in the nasal turbinate and respiratory tract. Although lesions were confirmed in the heart (pericardial effusion), liver, kidney, spleen, and lymph nodes, direct evidence of SARS-CoV-2 infection among these tissues was not observed [17]. These findings implied that immunopathology may be more important than local infection in the roles of multiple organ failures.

## Host innate and adaptive immune responses

Clinically, the host immune responses to SARS-CoV-2 infection are imbalanced, complicated, and varied over time [6,24,25]. Some severe cases showed strengthened inflammatory responses and uncontrolled cytokine release, indicating a high risk of cytokine storm and robust tissue injury not only in lung, but also systematically [24]. However, numbers of natural killer cells, T- and B-lymphocytes, as well as the percentage of monocytes, eosinophils, and basophils were drastically reduced in other severe cases. These cases usually have higher viral shedding, disease severity, and poor clinical outcome [26]. Recently, the SARS-CoV-2 receptor, ACE2, was demonstrated as an interferon-stimulated gene in human airway epithelial cells [27]. After SARS-CoV-2 infection, the expression of ACE2 was up-regulated by excessive expressed interferons [27]. In a recent clinical investigation of SARS-CoV-2 reinfection [28], the investigators compared the patients’ symptoms to those of their first infection and found that 68.8% of the convalescent patients had similar severity, 18.8% had worse symptoms, and 12.5% had milder symptoms, suggesting a risk of infection enhancement and more complicated virus-host interplay. As for adaptive immunity, a significant production of IgM and IgG antibodies is observed in patients. However, two clinical studies reported that the severe cases had an increased IgG response and a higher titre of total antibodies than the mild cases [29,30]. Meanwhile, a recent study showed an infectivity-enhancing site on the SARS-CoV-2 spike protein targeted by antibodies [31]. Collectively, these findings revealed a potential antibody-dependent enhancement (ADE). Therefore, whether the antibody response is protective or pathogenic needs to be determined in NHP models.

In the rhesus and cynomolgus macaques of SARS-CoV-2 infection, swollen mesenteric lymph nodes, increased number of T- and B-lymphocytes, and proinflammatory cytokines were detected [17], suggesting a robust host immune response. Meanwhile, significant hematological changes including neutrophils, lymphocytes, monocytes, haematocrits, red blood cells, haemoglobin, and reticulocytes were observed in another rhesus macaque’s study [21]. Importantly, the functional neutralizing antibody (NAb) was detectable from 10 to 12 dpi [17,21] and spike-specific NAb can be detected at 14 dpi [18]. Re-challenge of SARS-CoV-2 failed to cause diseases in three rhesus macaques recovered from initial infection [18], suggesting that vaccine or antibody-based therapy might be effective. Although ADE was not observed in the three re-infected rhesus macaques, this needs to be further assessed in more NHP models with a larger number, as well as large-scale randomized controlled clinical trials. Therefore, functional analysis and quantification of serum antibody by ELISA and cell-based functional tests [30,32–34] are essential for evaluating the ADE.

In summary, three points are extracted here from the studies of the interaction between the NHPs and SARS-CoV-2. First, anomalous host immune responses may lead to tissue damage, locally or systemically. Secondly, host factors including cellular receptor (ACE2), cytokines, chemokines, and interferons may play important roles to enhance or suppress infection and pathogenesis. Finally, high NAb titre in serum is adequate to prevent transmission.

## Evaluating vaccines and drugs in NHPs

After the outbreak of SARS-CoV-2, more than one hundred vaccines and drugs are currently under development worldwide [35]. An appropriate animal study is necessary to test the *in vivo* safety and effect of vaccines and drugs. The NHP model has been used for testing the efficiency of SARS-CoV-2 vaccines generated by different technologies including inactivation [36–38], expression of subunits such as spike protein or receptor-binding site (RBD) [39,40], DNA [41,42], mRNA [43–45], attenuated viral carrier [46–48], as well as drugs with different mechanisms [49–52]. The details of the preclinical NHP studies on some representative SARS-CoV-2 vaccines were summarized in Table 2. In the early stage of the SARS-CoV-2 pandemic, the inactivated vaccines have been rapidly developed and evaluated in rhesus macaques. In contrast to the placebo and control groups, viral RNA was over 100-fold lower in the throat and anal swabs, and undetectable in the lung of the high-dose vaccinated rhesus macaques [36]. Furthermore, no significant lung pathogenesis was observed in the vaccinated rhesus macaques, suggesting an adequate protection effect [36]. The ratio of T-lymphocytes in peripheral blood and concentrations of proinflammatory cytokines such as TNF-α, IFN-γ, and IL-2 were stable throughout the vaccination and virus challenge course for 30 days, revealing an absent of ADE [36]. The efficient protective immunity of mRNA vaccines was also well-demonstrated in NHP models [43–45]. In a rhesus macaque model of SARS-CoV-2 infection, mRNA-1273 vaccine candidate induced high NAb level, type 1 helper T cell (Th1) biased CD4-positive T cell responses and low or undetectable Th2 or CD8-positive T cell responses [44]. Viral replication was not detectable in lung tissues at 2 dpi [44]. Collectively, it was demonstrated by the NHP model that the inactivated vaccines and the mRNA vaccines are safe and effective in protecting NHPs from SARS-CoV-2 infection and severe pneumonia.

**Table 2.** Research details for preclinical NHP studies of representative SARS-CoV-2 vaccines derived from different technology roadmaps.

Research groups and NHP study of vaccines	Corbett et al.	Yang et al.	Yu et al.	Gao et al.	Wang et al.	Yang et al.	Sanchez-Felipe et al.	Mercado et al.
	mRNA vaccine		DNA vaccine	Inactivated vaccine		Subunit vaccine	Attenuated viral carrier vaccine	
Identifier and targets	mRNA-1273 (spike)	SW0123 (spike)	S(spike)/S.dCT/S.dTM/S1/RBD	PiCoVacc (mix)	BBIBP-CorV (mix)	RBD (aa319–545)	YF-S0 (yellow fever virus 17D vectored spike)	Ad26.COV2.S (adenovirus serotype 26 vectored spike)
Species	Rhesus macaques		Rhesus macaques	Rhesus macaques		Rhesus macaques	Cynomolgus macaques	Rhesus macaques
Gender and age and amount	12 of each sex; age range; 3–6 years	4 (sex and age were unknown)	35 male and female (6-12 years)	20 (sex not clear; 3–4 years)	10 (sex not clear; 3–4 years)	12 (sex not clear; 5–9 years)	12 mature male	52 male and female (6-12 years)
Vaccination dose	10/100 μg two doses	200 μg three doses	5 mg two doses	3/6 mg three doses	2/8 mg two doses	20/40 μg two doses	1×10E5 PFU two doses	1×10E11 viral particles one dose
Infection dose	7.5 × 10E6 PFU	1 × 10E6 PFU	1.1 × 10E4 PFU	1 × 10E6 TCID50		5 × 10E5 PFU	1.5 × 10E4 TCID50	1.1 × 10E4 PFU
Infection route	Intratracheal and intranasal		Intratracheal and intranasal	Intratracheal		Intranasal	Intratracheal and intranasal	
Protective effect	Viral shedding in respiratory tract, viral replication and inflammation in lung are rarely detectable by 2 dpi	Viral RNA is undetectable in respiratory tract and lung tissues at 7 dpi; SARS-CoV-2 induced lung pathological lesions were restored	Viral shedding in respiratory tract is suppressed by the five vaccines in varied degrees from 0 to 14 dpi	Viral shedding in respiratory tract, viral replication and inflammation in lung are suppressed from 0 to 7 dpi with a dose-dependent manner		Viral shedding in respiratory tract, viral replication and inflammation in lung are suppressed from 0 to 7 dpi	Viral shedding in respiratory tract was suppressed from 0 to 4 dpi	Viral shedding in respiratory tract is suppressed by the Ad26.COV2.S from 0 to 10 dpi
Clinical translation	Approved	Preclinical study	Preclinical study	Approved (over 16 billion shots applied)		Clinical trail	Preclinical study	Clinical trail

In order to inhibit viral replication and restore lung injury in the patients infected with SARS-CoV-2, drugs were developed and evaluated in animal models. Remdesivir (GS5734), a broad spectrum antiviral nucleotide prodrug, showed anti-MERS-CoV activity in cell culture study, mice, and rhesus macaques [53,54], and was further tested on rhesus macaques with SARS-CoV-2 infection [55]. Intravenous remdesivir treatment significantly reduced clinical symptom and chest X-ray scores, decreased virus titres in bronchoalveolar lavage fluid, and restored severe pneumonia in rhesus macaques. The lipoglyco-peptide antibiotic Dalbavancin can block the interaction between SARS-CoV-2 spike protein and host receptor ACE2, suppress viral replication and alleviate severe pneumonia in both hACE2 transgenic mice and rhesus macaques [49]. The (β-gal)-activated prodrug SSK1 restores severe pneumonia in rhesus macaques by down-regulating the SARS-CoV-2-induced proinflammatory cytokines such as IFN-γ, IL-6, IL-8, and IL-10 [50]. The NAb targets RBD on the spike protein showed prominent efficiency of preventing and treating SARS-CoV-2 infection in rhesus macaques [51,52].

## Discussion

The worldwide COVID-19 pandemic has been persisting for more than one year. However, many fundamental questions regarding SARS-CoV-2 infection and its pathogenesis are still unclear. Present studies have demonstrated that the NHP is a state-of-art animal model for investigating SARS-CoV-2 infection and its pathogenesis. Although the NHP models are available to mimic SARS-CoV-2 infection in humans and to exhibit typical symptoms and disease progress, some important potential effectors including age, gender, species, and host immune statues need to be tested in a larger sample size so that a statistical evaluation can be performed to draw a robust conclusion. Moreover, some obstacles exist in the translation of preclinical NHP studies to clinical trials. First, the experimental results from NHP models cannot always predict clinical outcomes. For instance, the antiviral drug remdesivir showed well-demonstrated therapeutic efficacy against SARS-CoV-2 in the rhesus macaques [55] but a limited clinical benefit for COVID-19 patients [56]. However, the mRNA vaccine is demonstrated to establish protective immunity in both the NHP models [44] and humans [57]. These varying results are caused by many factors. The mRNA vaccine can induce neutralizing antibodies and poly-specific T cells to eliminate the infectious viral particles directly, which were also recognized as indicators of disease severity [58]. In brief, the humans and NHPs with high NAb and robust T cell responses showed stronger immunity to SARS-CoV-2 infection and milder disease progress. Additionally, dynamic changes of these two indicators were detectable and similar in both the NHP models and humans. However, remdesivir cannot directly reduce the infectious viral particles. Furthermore, the lack of a clinical indicator limits its translation from NHP models to clinical trials. Secondly, the new emerging SARS-CoV-2 variants may cause breakthrough infection in vaccinated populations and enhanced disease, which necessitates an urgent development of optimized vaccines and drugs. Unique mutations can impact the infectivity, transmission capacity, and pathogenicity of SARS-CoV-2 variants [59–61]. The new emerging variants showed enhanced infectivity and transmission capacity [62–64], as well as strong resistance to the convalescent serum and vaccine-induced NAb [65–67]. These findings revealed that the protection efficiency of RBD-derived vaccines might be decreased by the emerging of SARS-CoV-2 variants. Therefore, a systematic evaluation of typical SARS-CoV-2 variants in NHP and other animal models will be helpful to understand viral evolution and find out the critical mutation sites. More importantly, to know the “off-target” effect of current vaccine and drugs on the new emerging SARS-CoV-2 variants, a rapid efficiency analysis in an animal model is urgently in need. Thirdly, several unique clinical symptoms such as asymptomatic infection [68,69], vomiting, diarrhoea and gastrointestinal infection [70,71], venous thromboembolism, lymphopenia [72], and sepsis were recently reported in human patients. However, these symptoms were rarely observed in NHP studies. In addition, patients with diseases such as cancer, diabetes, cardiovascular diseases, and chronic virus infections showed a higher severity and mortality after SARS-CoV-2 infection [6]. Therefore, the evaluation of combined therapy strategies that combat both SARS-CoV-2 and these diseases in animal models are necessary for clinical therapy of these high-risk populations.

Although the NHPs have been recognized as ideal animal models to study human infectious diseases, their application was largely limited by several drawbacks. For instance, strong ethic restrictions, high costs, relatively low breeding efficiency, long-term maturation, large body size, complicated operations and individual variation among NHPs make it difficult to conduct research in a large sample size. Moreover, the NHP models for pathogens with highly infectivity (such as SARS-CoV, SARS-CoV-2, and MERS-CoV) should be operated in ABSL-3 laboratory and isolation devices throughout the whole infection course. The NHP models and facilities at hand are inadequate to meet the huge demand, which might impede our further understanding of the mechanisms of SARS-CoV-2 pathology, protective immunology, and drug therapy, and limit further translation of vaccines and drugs. Indeed, the development of many vaccines and drugs was blocked in the stage of preclinical animal study.

Fortunately, advances in technologies include scalable NHP clones, stable gene modification, rapid passage, and maturation provide efficient approaches to optimize SARS-CoV-2-infected NHP models. Liu et al. recently reported the accelerated passage of gene-modified cynomolgus macaques by hormone-induced precocious puberty [73]. By using the NHP cloning technology, the investigators can rapidly achieve a scalable production of standardized laboratory NHP strains with relatively low individual diversity than the NHPs generated by traditional methods. Moreover, the gene modification technology is useful to identify and verify the roles of host genes in the process of SARS-CoV-2 infection and pathogenesis. For instance, the NHP modified with genes that enhance SARS-CoV-2 infection and pathogenesis might better resemble human patients with high viral load and severe respiratory symptoms. Collectively, the optimization of NHP models will expand their application and translation in the future studies of SARS-CoV-2 and new emerged pathogens. Overall, we should keep the high experimental standards and strict operation to ensure that the safety and effectiveness of each tested vaccine or antiviral (anti-inflammatory) agent are accurately displayed in the experimental animals.
